# Queen–worker ratio affects reproductive skew in a socially polymorphic ant

**DOI:** 10.1002/ece3.1779

**Published:** 2015-11-17

**Authors:** Bartosz Walter, Jürgen Heinze

**Affiliations:** ^1^ Museum and Institute of ZoologyPolish Academy of SciencesWilcza 6400‐679WarsawPoland; ^2^ Biologie IUniversität RegensburgD‐93040RegensburgGermany

**Keywords:** Dominance, *Leptothorax*, polygyny, reproductive potential, reproductive skew

## Abstract

The partitioning of reproduction among individuals in communally breeding animals varies greatly among species, from the monopolization of reproduction (high reproductive skew) to similar contribution to the offspring in others (low skew). Reproductive skew models explain how relatedness or ecological constraints affect the magnitude of reproductive skew. They typically assume that individuals are capable of flexibly reacting to social and environmental changes. Most models predict a decrease of skew when benefits of staying in the group are reduced. In the ant *Leptothorax acervorum*, queens in colonies from marginal habitats form dominance hierarchies and only the top‐ranking queen lays eggs (“functional monogyny”). In contrast, queens in colonies from extended coniferous forests throughout the Palaearctic rarely interact aggressively and all lay eggs (“polygyny”). An experimental increase of queen:worker ratios in colonies from low‐skew populations elicits queen–queen aggression similar to that in functionally monogynous populations. Here, we show that this manipulation also results in increased reproductive inequalities among queens. Queens from natural overwintering colonies differed in the number of developing oocytes in their ovaries. These differences were greatly augmented in queens from colonies with increased queen:worker ratios relative to colonies with a low queen:worker ratio. As assumed by models of reproductive skew, *L. acervorum* colonies thus appear to be capable of flexibly adjusting reproductive skew to social conditions, yet in the opposite way than predicted by most models.

## Introduction

Group‐living and communal breeding in animals are typically associated with conflict about the partitioning of reproduction among group members. This conflict may be resolved by the formation of rank orders, in which dominant individuals suppress subordinate reproduction and gain a disproportionally large share in reproduction (“high reproductive skew”, (Keller and Reeve 1994; Vehrencamp 1983; Johnstone 2000)). On the contrary, reproductive skew among queens in multiqueen societies of ants is usually low: Queens appear to widely ignore each other's presence and contribute similarly to brood production (polygyny; (Heinze 1993; Keller 1995; Haag‐Liautard et al. 2008)). High or even maximal skew, in which a single, socially dominant queen monopolizes reproduction, is known only from a minority of ants (functional monogyny, (Buschinger 1968; Heinze and Smith 1990; Yamauchi et al. 2007)).

The magnitude of reproductive skew varies tremendously among populations of the ant *Leptothorax acervorum* (Heinze et al. 1995; Gill et al. 2009). Queens in colonies from extended forests in central and northern Eurasia do not form rank orders and on average have similar egg laying rates (Buschinger 1968; Hammond et al. 2006). In contrast, queens from populations at the margins of the species' distribution engage in ritualized fights (Heinze and Ortius 1991; Ito 2005; Trettin et al. 2011) or are unequally treated by workers (Gill and Hammond 2011), which results in a dominance hierarchy in which only the top‐ranking queen lays eggs. This variation is suggested to be associated with different degrees of environmental harshness and constraints on dispersal and solitary colony founding (Bourke and Heinze 1994).

Skew models assume that individuals can react plastically to changes in their environment (Kokko 2003) and predict that skew should decrease if benefits of group‐living are reduced (Nonacs and Hager 2011). Recently, it was shown that the behavior of *L. acervorum* is not fixed within populations and that an increase of queen:worker ratio can elicit aggression among normally peaceful queens from a low‐skew population (Trettin et al. 2014). Unfortunately, the consequences of this manipulation for reproductive skew were not evaluated, calling for a more detailed examination. Here, we expanded the previous experiment concentrating on measures of fecundity. We show that increased queen:worker ratio leads to increased reproductive skew. Thus, colonies appear to be capable of flexibly adjusting reproductive skew to social conditions, yet in the opposite way than predicted by most models (for review, see Nonacs and Hager (2011)).

## Material and Methods

**Figure 1:**
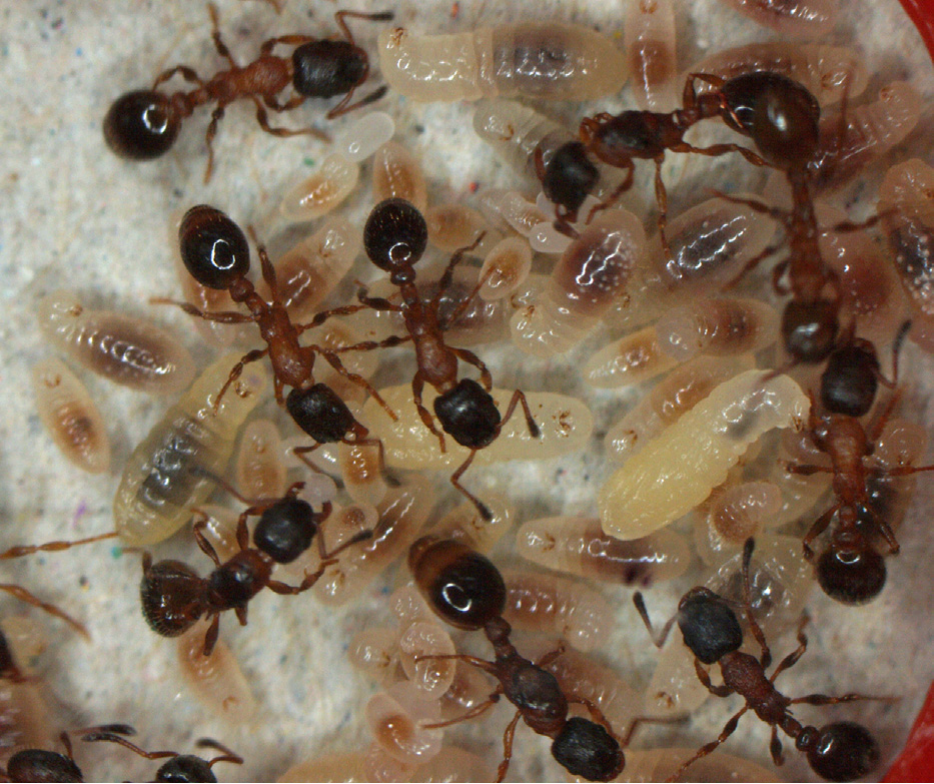
Polygynous colony of the ant *Leptothorax acervorum* with three queens, workers, larvae, and eggs.

We collected 71 overwintering colonies of *Leptothorax acervorum* in Otwock, Poland, on 15 February 2014. Of these, 21 consisted of two or more inseminated queens (polygynous colonies) with 8 to 79 workers per queen (mode = 18 workers/queen). Only these polygynous colonies were further investigated (see Fig. 1). Queens from seven overwintering colonies were dissected right after collecting (later referred to as “overwintering queens” from “natural overwintering colonies”). Overwintering queens in each colony were ranked starting from “the most fecund” to “the least fecund” queen based on the total number of oocytes larger than 50 *μ*m in diameter present in their ovaries. In a second group of seven colonies, we standardized the queen:worker ratio to 18 workers per queen by removal of surplus workers (“low queen:worker ratio”: queens 2–6; workers: 36–108). In a third group of seven colonies, we reduced the number of workers to four per queen (“high queen:worker ratio”: queens 2–6; workers: 8–24) to check whether queen:worker ratio has an influence on reproductive skew. Initial worker numbers did not differ before manipulation (“natural overwintering”: 30–109, median 81; “low queen:worker ratio”: 36–157, median 74; “high queen:worker ratio” 24–134, median 54; Kruskal–Wallis test *H* = 2.06, *P *>* *0.3). Queen:worker ratios of 1:4 or more are commonly observed in high‐skew populations of *L. acervorum* (e.g., see figure S1 in Trettin et al. 2014). For example, the ratio varied from 1:8 to 1:4 queens per worker in *L. acervorum* colonies in Japan (Ito 2005), and queen:worker ratios ≥1:4 were observed in 25 of 50 colonies from Spain (Felke 1999; Felke and Buschinger 1999). Therefore, the ratio employed in our study is biologically realistic and resembles natural conditions observed in high‐skew populations.

**Figure 2:**
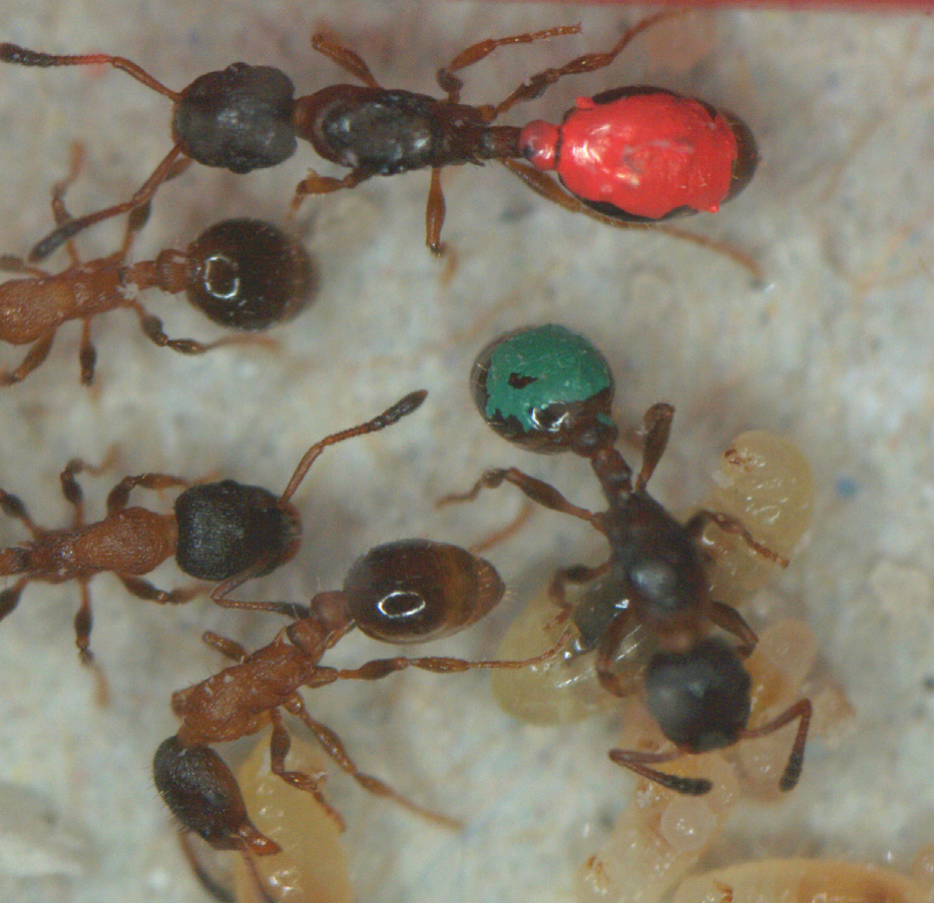
Queens of the ant *Leptothorax acervorum* individually marked with paint.

All queens were individually marked (see Fig. 2), and colonies were cultivated as described in (Heinze and Ortius 1991) in artificial spring conditions (12 h day/night, +18/+15°C). Queen behavior was observed for 15 consecutive days starting on the second day after collection (300 min/queen, two 10‐min observations/day). We noticed the time spent by queens on the brood pile, aggression, and grooming. As no eggs had yet been laid within this period, we monitored queens for additional 15 days for 1 min per day. Thereafter, the queens were frozen and their ovaries were dissected. Oocytes are produced in the posterior end of tube‐like filaments (ovarioles) and, as they mature, increase in size, and are relocated toward the ovariole base. Following (Dolezal et al. 2013) we divided the developing oocytes into vitellogenic oocytes (opaque) and previtellogenic oocytes (translucent). To determine the queen's fecundity, we counted all oocytes larger than 50 *μ*m in diameter, clearly separated by nurse cells (“total number of oocytes”). Nestmate queens were ranked according to the number of vitellogenic oocytes and given the labels “most fecund queen,” “second most fecund,” etc. until “least fecund queen.” Colonies with two queens had only “the most fecund queen” and “the least fecund queen.” Reproductive skew was estimated from the number of oocytes using Nonacs' B‐index (Nonacs 2000) and the software Skew Calculator 2003 (https://www.eeb.ucla.edu/Faculty/Nonacs/pdfs/skewcalculatormanual.pdf). Generalized linear model (GLZ) with gamma distribution of data (dependent variable did not have a normal distribution) and logarithmic function used as a linking function was built to test effects between B‐indices (dependent variable) and queen number, worker number, and queen:worker ratio as covariates in Statistica 6.0 (Tulsa, OK).

## Results

**Figure 3:**
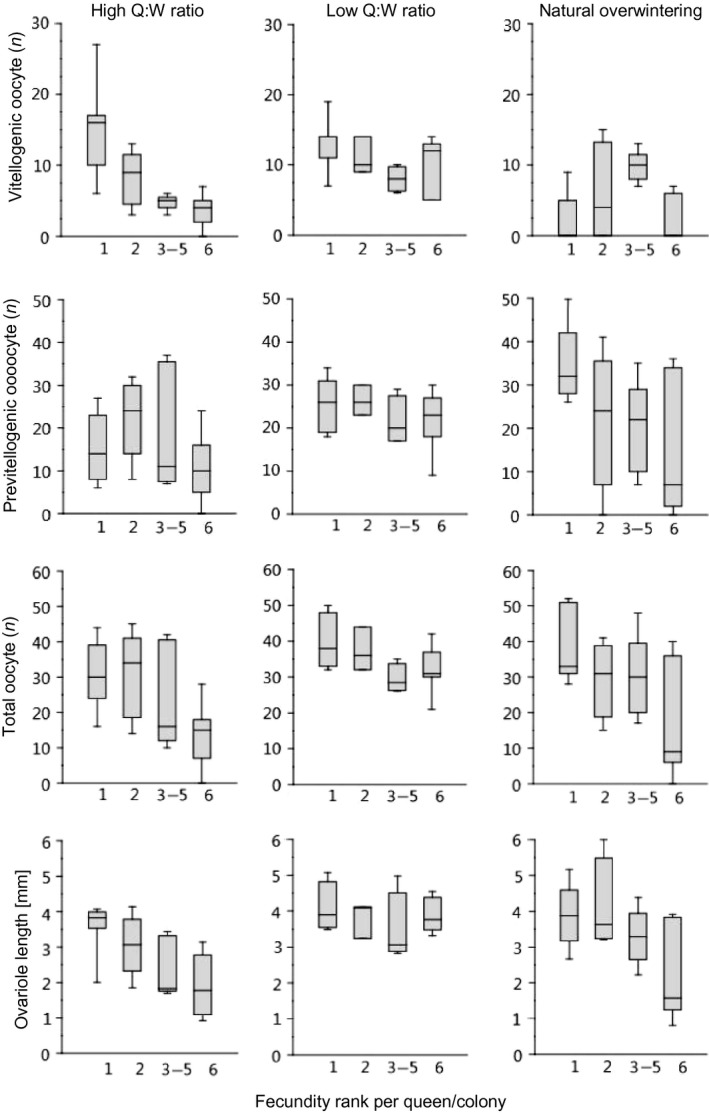
Differences in ovary development among queens of the socially plastic ant *Leptothorax acervorum* from experimental colonies with a high (1:4) queen:worker ratio, colonies with a low (1:18) queen:worker ratio, and natural overwintering colonies. Vitellogenic oocytes are maturing oocytes that develop into eggs.

Overwintering queens varied strongly in the total number of oocytes in their ovaries, which can be a proxy for their current fecundity potential (difference among groups of nestmate queens ranked according to oocyte number: Kruskal–Wallis test, *H = *9.10, *P *<* *0.011, Fig. 3). As expected from our a priori ranking of queens according to fecundity, the most fecund queens had on average three times more oocytes than the least fecund queens (post hoc test: *P *<* *0.0054, corrected for multiple comparisons: *P*′ < 0.0216). Differences in the number of maturing vitellogenic oocytes were insignificant (*H = *6.54, *P *=* *0.09) as queens normally cease their production while overwintering. Overwintering queens had ovarioles of similar length (*H *=* *3.3, *P *=* *0.34). Ovariole length was also similar among queens in colonies with a low queen:worker ratio (*H *=* *3.07, *P *=* *0.38). In contrast, ovariole length differed among queens from colonies with high queen:worker ratio (*H *=* *11.75, *P *<* *0.001, Fig. 3), where the most fecund queens had significantly longer ovarioles than all other nestmate queens (all cases: *P *<* *0.02; 0.04 < *P*′ < 0.09).

The total numbers of previtellogenic and vitellogenic oocytes varied similarly among queens in both colonies with low (*H *=* *10.52, *P *<* *0.006) and high queen:worker ratio (*H *=* *8.04, *P *<* *0.018; Fig. 3). As a trivial consequence of our ranking of queens according to fecundity, the difference was most pronounced between queens with highest and least fecundity (low queen:worker ratio: *P < *0.004, *P′ *< 0.016; high queen:worker ratio: *P *<* *0.007, *P′ *< 0.029). Importantly, despite of similar ranking, the number of vitellogenic oocytes in the ovaries differed only among nestmate queens in colonies with a high queen:worker ratio (high ratio: *H *=* *14.55, *P *<* *0.003). Here, the ovaries of the most and the second most fecund queens contained similar numbers of vitellogenic oocytes (*P′ *= 0.51), but significantly more than those of less fecund queens (all cases *P*′ < 0.05, Fig. 3). In contrast, no significant differences were found among the similarly ranked nestmate queens in colonies with a low queen:worker ratio (*H *=* *5.97, *P *=* *0.1). This indicates that fecundity varies more among nestmate queens from colonies with a high queen:worker ratio than among queens from colonies with a low queen:worker ratio due to diminished fecundity of least fecund queens in colonies with high queen:worker ratio. The most fecund queens from colonies with low and high queen:worker ratio had similar numbers of vitellogenic oocytes (Mann–Whitney *U*‐test, oocytes total: *U *=* *12.0, *P = *0.12; vitellogenic: *U *=* *22.0, *P = *0.80). The least fecund queens from colonies with a high queen:worker ratio were considerably less fecund than the least fecund queens from colonies with low queen:worker ratio (*U *=* *4.0, *P < *0.02).

**Figure 4:**
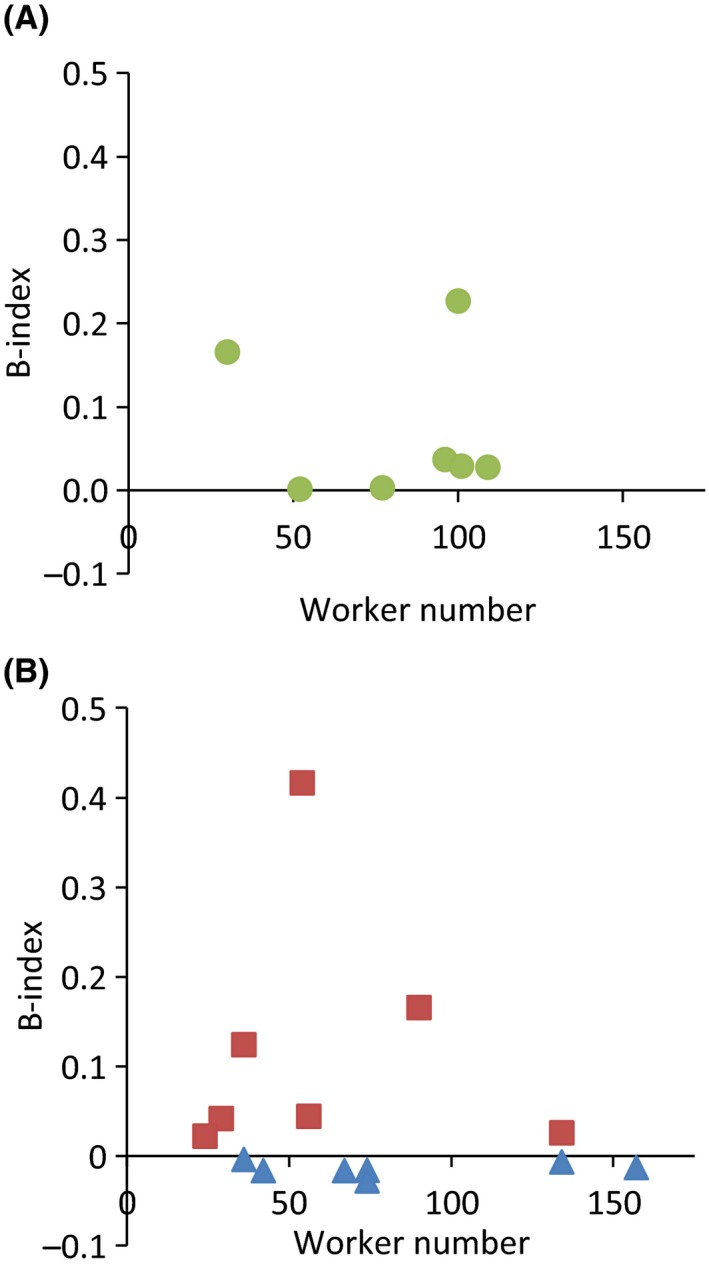
Skew in relation to queen:worker ratio in colonies of the ant *Leptothorax acervorum*. Increased queen:worker ratios led to increased reproductive skew. Positive B‐indices indicate that reproduction is more skewed, negative B‐indices that reproduction is less skewed than expected from random variation. (A) natural overwintering colonies, B‐indices measured for number of previtellogenic oocytes. (B) experimental colonies with low (blue triangles) and high (brown squares) queen:worker ratios, B‐indices measured for number of vitellogenic oocytes.

Reproductive skew was low in low queen:worker ratio colonies as measured by B‐indices for the number of vitellogenic oocytes (all cases *P *>* *0.47, Fig. 4, Table S1). In contrast, reproductive skew was higher than expected in five of seven colonies with high queen:worker ratio (*P *<* *0.04, Fig. 4, Table S1). B‐indices were higher in colonies with a high queen:worker ratio than in colonies with a low queen:worker ratio (Mann–Whitney *U*‐test *P *<* *0.003; Fisher exact *P *=* *0.021). A model with B‐indices as dependent variable and queen and worker numbers as covariates showed a significant effect of queen:worker ratio on skew (Wald statistic: queen:worker ratio *W* = 4.29, *P *<* *0.04). The effects of covariates were insignificant (worker number *W* = 1.69, *P *=* *0.19; queen number *W* = 3.29, *P *=* *0.07).

Reproductive skew measured for vitellogenic oocytes was moderately correlated with the absolute number of workers after manipulation (gamma = −0.43, *P *<* *0.05) and uncorrelated with the number of queens (gamma = 0.36, *P *=* *0.11). Across colonies, the number of vitellogenic oocytes of the most fecund queens increased with increasing number of workers (gamma = 0.75, *P *<* *0.04) and queens (gamma = 0.75, *P *<* *0.04) in colonies with high queen:worker ratio but not so in colonies with low queen:worker ratio (all cases: *P *=* *0.18). The fecundity of less fecund queens did not correlate with worker or queen number in any of two colony types (in all cases *P *>* *0.1).

**Figure 5:**
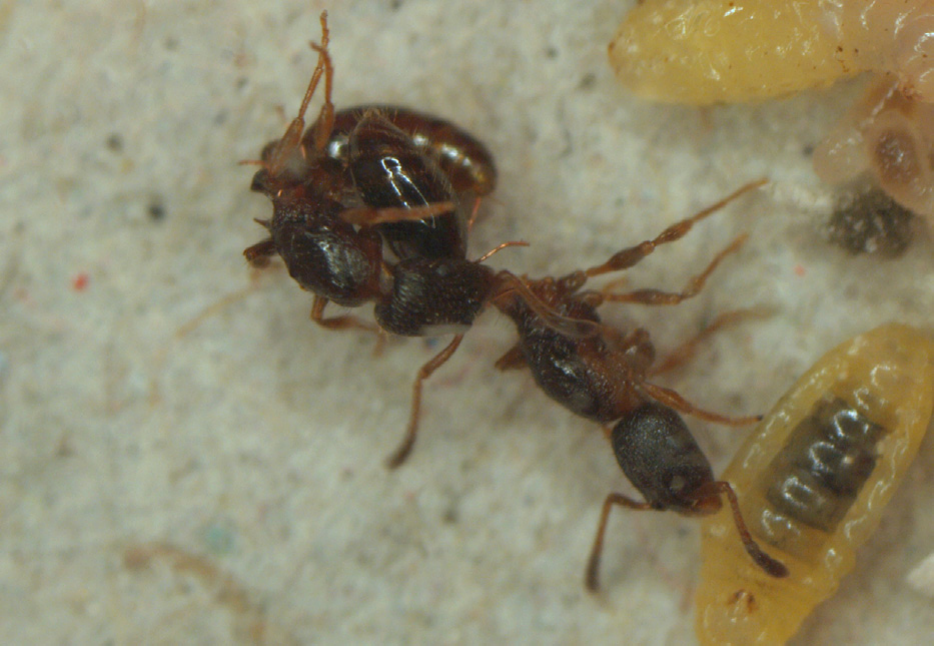
Two queens of the ant *Leptothorax acervorum* fighting for dominance.

In colonies with low queen:worker ratio, queens of different fecundity received similar amounts of grooming and spent similar time on the brood pile (all cases *P *>* *0.1). In colonies with a high queen:worker ratio, the most fecund queens appeared to receive more grooming than less fecund queens, but these differences were not significant after Bonferroni correction for multiple comparisons (*P*′ > 0.2 in all cases). The absolute number of workers moderately affected grooming duration received by queens of any rank in colonies with high queen:worker ratio (gamma = −0.35, *P *<* *0.04). In colonies with low queen:worker ratio, queens of different rank did not differ in time of received grooming from workers (*H* = 0.52, *P *>* *0.9) and absolute number of workers did not affect grooming duration received by queens (gamma′0.05, *P *>* *0.8). Overt aggression was observed in two colonies with low queen:worker ratio and four colonies with high queen:worker ratio (Fig. 5), but the low number of interactions and their short duration did not allow a statistical test.

## Discussion

We found that a drastic increase of queen:worker ratios by worker removal increases reproductive skew among queens in colonies of the socially polymorphic ant *Leptothorax acervorum* from a low‐skew population. This matches earlier observations that worker removal leads to queen–queen antagonism (Trettin et al. 2014) and corroborates the hypothesis that queens are capable of adjusting their reproductive strategies in response to social changes (Trettin et al. 2014). This supports the assumptions of reproductive skew models that cooperative individuals are capable of accommodating skew in relation to ecological constraints (Vehrencamp 1983; Johnstone 2000; Nonacs and Hager 2011). Yet, most skew models predict that our manipulation should have diminished reproduction by the most fertile queens and not by less fertile queens. When the reproduction of a group decreases, subordinates should keep their level of reproduction to prevent them from leaving the group (e.g., Buston and Zink 2009). The discrepancy might be explained by the inability of queens to leave our experimental nests (Nonacs and Hager 2011). Reproductive skew may thus be a plastic trait, as assumed by optimal skew models, rather than genetically fully fixed among populations (Kokko 2003). Field studies indeed show that skew may vary both in polygynous and functionally monogynous colonies (Hammond et al. 2006; Trettin et al. 2011). It remains to be studied whether, for example, colonies with above‐average skew in polygynous populations have a more unbalanced queen‐worker ratio, suffer more severe resource shortening, or contain queens that by chance differ more strongly in their innate fecundity than in colonies with a more balanced partitioning of reproduction.

Another interesting question that arose from our results is whether high skew would have persisted if we had continued the experiment. One possible scenario is that the least fertile queens would have been eliminated from the high‐skew colonies, as they constitute substantial costs without providing benefits. The colonies would then revert to low skew among the remaining queens. Alternatively, the least fecund queens might have remained in the colonies and started reproduction later in the season once the colonies had grown and re‐established optimal queen‐worker ratios. In such cases, low skew would be the long‐term evolutionary stable strategy (ESS) for all colonies, independent of immediate ecological constraints or queen–worker ratios. Alternatively, reproduction might have remained highly skewed, but reproductive rank orders might have changed and less fecund queens might have superseded the formerly more productive queens. These possibilities will be explored in future studies. In any case, the retention of population‐specific social structure under prolonged laboratory culture suggests that queens from different populations may have different thresholds when reacting to social or environmental disturbances (Heinze et al. 1995; Gill et al. 2009).

Overwintering queens differed in the number of developing oocytes, perhaps reflecting differences in age, previous reproductive experience, and innate variation in ovarian activity (e.g., Shukla et al. 2013). These differences might be amplified under high queen:worker ratios, probably through queen–queen aggression, worker–queen aggression, and different levels of worker care, as observed in high‐skew populations of *Leptothorax* (Heinze and Smith 1990; Heinze and Ortius 1991; Ito 2005; Gill et al. 2009; Gill and Hammond 2011; Trettin et al. 2011). The manipulation of queen:worker ratios mainly decreased the fecundity of the least fertile queens. The ovaries of the most fertile queens from colonies with high and low queen:worker ratio contained similar numbers of oocytes. In contrast, the ovaries of the least fertile queens from colonies with a high queen:worker ratio contained only half the number of oocytes than those of the least fertile queens from colonies with a low queen:worker ratio. Colonies might thus be capable of adjusting brood production by multiple queens to environmental changes and to avoid an over‐production of eggs that cannot all be reared to adulthood.

National Science Centre provided funding (decision DEC‐2011/01/B/NZ8/05951 to BW). We thank Peter Nonacs and three anonymous referees for comments and K. Zielska and M. Brouze for assistance.

## Conflict of Interest

None declared.

## Supporting information


**Table S1.** Skew and queen:worker ratio in colonies of the ant *Leptothorax acervorum*.
