# Supplementary material for: Queen–worker ratio affects reproductive skew in a socially polymorphic ant
Source: Ecol Evol. 2015 Nov 17;5(23):5609–15. doi: 10.1002/ece3.1779 (PMC4813118; doi:10.1002/ece3.1779)
Supplement: Supplementary file 1 — Table S1. Skew and queen:worker ratio in colonies of the ant Leptothorax acervorum. [file ECE3-5-5609-s001.docx]

Supplementary Table 1. Skew and queen:worker ratio in colonies of the ant *Leptothorax acervorum*. Reproductive skew as increased in colonies with high queen:worker ratio (1/4) compared to colonies with low queen:worker ratio colonies (1/18). Positive B-indices indicate that reproduction is more skewed, negative B-indices that reproduction is less skewed than expected from random variation. Bold font indicates colonies with skew significantly differed from zero.

| Colony name | Queens/colony | B-index | P | CIs |
| --- | --- | --- | --- | --- |
| 1 low queen:worker ratio | 3 | -0.0159 | 1 | -0.0163; 0.0003 |
| 2 low queen:worker ratio | 4 | -0.0128 | 0.83 | -0.0179; 0.0069 |
| 3 low queen:worker ratio | 2 | -0.0278 | 0.79 | -0.0417; 0.0832 |
| 4 low queen:worker ratio | 6 | -0.0065 | 0.68 | -0.0177; 0.0122 |
| 5 low queen:worker ratio | 2 | -0.0163 | 0.84 | -0.0192; 0.0225 |
| 6 low queen:worker ratio | 2 | -0.0037 | 0.48 | -0.0152; 0.0427 |
| 7 low queen:worker ratio | 2 | -0.0163 | 0.84 | -0.0192; 0.0251 |
| 1 high queen:worker ratio | **6** | **0.1658** | **0.0001** | **0.1378; 0.3019** |
| 2 high queen:worker ratio | **3** | **0.1244** | **0.029** | **0.0594; 0.2188** |
| 3 high queen:worker ratio | **2** | **0.4167** | **0.033** | **0.1367; 0.4167** |
| 4 high queen:worker ratio | **5** | **0.0444** | **0.017** | **0.0283; 0.0794** |
| 5 high queen:worker ratio | 3 | 0.026 | 0.082 | 0.0126; 0.712 |
| 6 high queen:worker ratio | **3** | **0.0419** | **0.029** | **0.0225; 0.088** |
| 7 high queen:worker ratio | 2 | 0.0222 | 0.3 | -0.0333; 0.1432 |
